# Prognostic Value of LncRNA HOTAIR in Colorectal Cancer: A Meta-analysis

**DOI:** 10.1515/med-2020-0012

**Published:** 2020-02-11

**Authors:** Shuangqian Chen, Chunxiao Zhang, Maohui Feng

**Affiliations:** 1 Department of Gastrointestinal Surgery, Zhongnan Hospital of Wuhan University; Hubei Key Laboratory of Tumor Biological Behaviors, Wuhan 430071, China; 2 Department of Ultrasonography, Zhongnan Hospital of Wuhan University; Hubei Key Laboratory of Tumor Biological Behaviors, Wuhan 430071, China

**Keywords:** lncRNA HOTAIR, Colorectal cancer, Prognosis, Clinicopathological features, TCGA

## Abstract

Abstract: HOX transcript antisense intergenic RNA (HOTAIR) is one of the most studied long noncoding RNAs (lncRNAs) and is aberrantly expressed in colorectal cancer (CRC). We thus performed a comprehensive study based on meta-analysis and validation of the TCGA database to investigate clinicopathological and prognostic value of HOTAIR in CRC. Six studies enrolling 629 CRC patients were included in the analysis. The results indicated that high HOTAIR expression predicted worse OS (hazard ratio [HR] = 2.46, 95% confidence interval [CI]: 1.82-3.32, P < 0.01) and RFS (HR = 1.97, 95% CI: 1.27-3.05, P < 0.01) for CRC patients. High HOTAIR expression was also significantly associated with venous invasion (OR = 2.53, 95% CI: 1.12-5.68, P = 0.02), advanced tumor infiltration (OR = 3.35, 95% CI: 1.34-8.42, P = 0.01) and distant metastasis (OR = 5.52, 95% CI: 1.22-25.01, P = 0.03). Then, the results were validated by the TCGA database, showing that the up-regulated expression of HOTAIR was significantly related to poor OS (P = 0.01) and RFS (P = 0.04) in CRC. Our meta-analysis indicated that high HOTAIR expression was closely associated with poor clinical outcomes and could be a reliable prognostic biomarker for CRC patients.

## 1 Introduction

Colorectal cancer (CRC) is one of the most common cancers in the world [1]. Although surgery, chemoradiotherapy and targeted therapy make great progress to prolong survival of CRC patients, CRC is still the second leading cause of cancer related death worldwide, mainly attributed to tumor relapse [1,2]. Thus, it is vital to predict local recurrence and distant metastasis for improving prognosis in CRC. For decades, the tumor-node-metastasis (TNM) staging system is widely applied to predict prognosis and to guide treatment for CRC patients [3]. However, prognosis varies significantly in patients with same tumor stage due to individual heterogeneity [4]. This leaves a large space to search supplementary biomarkers for better predicting prognosis of CRC patients.

Long noncoding RNAs (lncRNAs) are broadly defined as RNA molecules greater than 200 nucleotides in length, and lacking important open reading frameworks [5, 6, 7]. They are regulators of gene expression at the chromatin-organizational, transcriptional and post-transcriptional levels [8]. Abundant evidence has demonstrated that lncRNAs play significant regulatory role in the process of proliferation, migration and invasion of tumor cells [9]. Currently, the relationship between the expression of particular lncRNAs and prognosis of cancer patients has also been increasingly reported, especially HOX transcript antisense intergenic RNA (HOTAIR), a highly oncogenic lncRNA in various malignancies [10,11].

HOTAIR was first identified as a polyadenylated RNA with 2,158 nucleotides and 6 exons, and expressed from the HOXC gene cluster locus on chromosome 12q13.13 [12]. Subsequently, more evidence had been accumulated about the aberrant expression of HOTAIR in various cancers, and the pivotal role in cancer progression and metastasis, such as lung cancer, gastric cancer and hepatocellular cancer [13, 14, 15]. Meanwhile, several studies have investigated the association between the development and metastasis of CRC and expression level of HOTAIR, showing that it is related to differentiation, distant metastasis and TNM stage in CRC patients [16,17]. However, whether its abnormal expression is correlated with prognosis for CRC patients remains inconclusive. Therefore, we aimed to get an entire understanding about HOTAIR and its expression level correlation with overall survival (OS), relapse-free survival (RFS) and clinicopathological features in CRC. The Cancer Genome Atlas (TCGA) database was employed to validate the prognostic value of HOTAIR in CRC.

## 2 Methods

### 2.1 Literature search

This study meets the guidelines of Preferred Reporting Items for Systematic Reviews and Meta-Analyses (PRISMA) [18]. We retrieved four databases (PubMed, Web of science, Embase and Cochrane library) for relevant studies that assessed the prognostic role of HOTAIR for CRC patients. The key words used for research were “long noncoding RNA” or “lncRNA”, “HOX transcript antisense intergenic RNA” or “HOTAIR” and “colorectal cancer” or “colon cancer” or “rectal cancer”. The last search ended on March 1, 2019. In addition, potential related studies were also searched in the references in the identified articles.

### 2.2 Study inclusion and exclusion criteria

Inclusion criteria for selecting the articles in our analysis were as follows: 1) the patients were diagnosed as CRC based on histopathological observation; 2) investigated the association between HOTAIR expression in CRC tissues and clinicopathological features and survival information; 3) hazard ratio (HR) with its 95% confidence interval (CI) were allowed or could be reconstructed by data reported [19]; 4) papers were published in English. Exclusion criteria were as follows: 1) reviews, case reports or laboratory studies; 2) studies without sufficient data for calculating HRs with its 95% CIs; 3) studies contained duplicate data.

### 2.3 Data extraction and quality assessment

Two authors independently assessed the potential studies based on the above-mentioned criteria. For each study, we extracted the following entries: 1) first author, year of publication, country, sample size, detection method, follow-up period and cut-off value; 2) clinicopathological features including tumor size, differentiation, venous invasion, tumor infiltration, lymph node metastasis, distant metastasis and TNM stage; 3) survival outcomes including OS and RFS. Quality assessment was evaluated by the Newcastle-Ottawa Scale (NOS) [20]. The NOS scores of ≥ 6 were considered as high-quality studies.

### 2.4 Validation in the TCGA database

This study was performed according to the publication guidelines supplied by TCGA (https://cancergenome.nih.gov/publications/publicationguidelines) Gene Expression Profiling Interactive Analysis (GEPIA, http://gepia.cancer-pku.cn) which analyzed gene expression based on RNA sequencing (RNA-Seq), was employed to analyze the TCGA database. Kaplan-Meier analysis was conducted to assess prognosis.

### 2.5 Statistical analysis

Statistical analyses were performed by Stata 12.0 software (STATA Corporation, College Station, TX, USA). HR with its 95% CI were obtained directly from included studies or estimated by survival curves. Odds ratio (OR) with its 95% CI were combined as the effective value to analyze the correlation between HOTAIR expression and clinicopathological parameters. Heterogeneity among pooled results was assessed by Cochran’s Q test and Higgins I-squared statistic. Sensitivity analysis was performed to examine the stability of results. Begg’s test and Egger’s test were used to assess the potential publication bias.

## 3 Results

### 3.1 Description of the included studies

**Figure 1:**
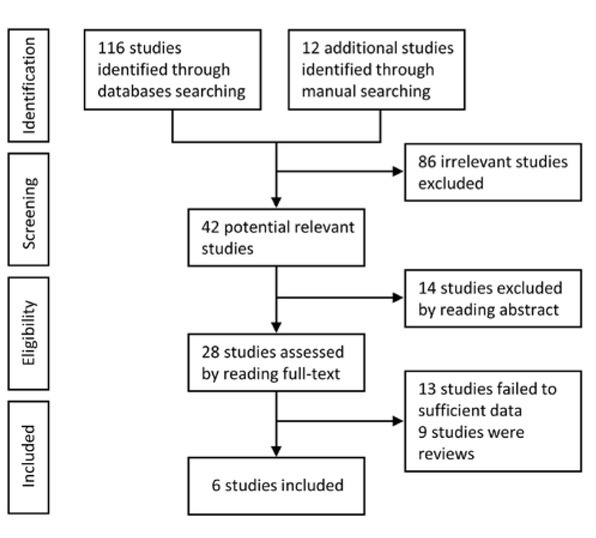
The flow chart of study selection.

According to the aforementioned search strategies, 128 studies were retrieved. By intensive reading of potential articles, 122 studies were excluded. Lastly, 6 studies which were published between 2011 and 2018 were included in this meta-analysis (Figure 1) [16,17,21,22,23,24]. Characteristics of included studies are summarized in Table 1. Six retrospective studies were included containing 629 CRC patients. Four studies were from China, one from Japan and one from Czech Republic. The sample size of included articles ranged from 73 to 152 CRC patients. Real-time quantitative

**Table 1:** Characteristics of included studies.

Study	Year	Country	Sample size	Male/ Female	Cut-off value	Detection method	Follow-up (months)	Survival analysis	NOS score
Kogo et al^16^	2011	Japan	100	63/37	H/G = 0.27	RT-qPCR	132	OS	7
Svoboda et al^21^	2014	Czech	73	46/27	median	RT-qPCR	54	OS	8
Wu et al^22^	2014	China	120	64/56	T/N > 5	RT-qPCR	72	OS, RFS	8
Luo et al^24^	2016	China	80	43/37	median	RT-qPCR	NR	OS	6
Li et al^17^	2017	China	152	105/47	ROC	RT-qPCR	70	OS, RFS	7
Xiao et al^23^	2018	China	104	63/41	median	RT-qPCR	60	OS	6

**Abbreviations:** NOS, Newcastle-Ottawa Scale; H/G, HOTAIR/GAPDH; T/N, tumor/normal; ROC, receiver operating characteristic; RT-qPCR, real-time quantitative PCR; OS, overall survival; RFS, relapse-free survival; NR, not reported.

PCR (RT-qPCR) was performed to measure the HOTAIR expression in CRC tissues. All studies were high-quality based on the NOS score.

### 3.2 Association between HOTAIR and survival outcome in CRC

**Figure 2:**
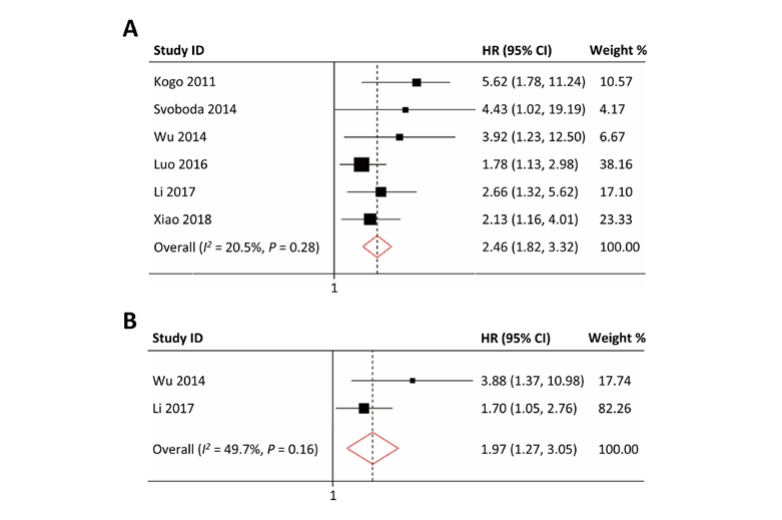
The forest plot between HOTAIR expression and survival outcome in colorectal cancer. (A) HOTAIR and overall survival. (B) HOTAIR and relapse-free survival.

Six studies reported the relationship between HOTAIR and OS, indicating that CRC patients with its high expression had significantly worse OS than those with its low expression (HR = 2.46, 95% CI: 1.82-3.32, *P* < 0.01) with no obvious heterogeneity (*I^2^* = 20.5%, *P* = 0.28) (Figure 2A).

Then we conducted subgroup analysis according to confounders such as country, cut-off value, sample size and NOS score (Table 2). Subgroup analyses by country indicated that high HOTAIR expression predicted poor OS for patients both in China (HR = 2.16, 95% CI: 1.56-2.98, *P* < 0.01) and in other countries (HR = 5.25, 95% CI: 2.41-11.47, *P* < 0.01). Stratification by cut-off value, we found the pooled HRs were 2.01 (95% CI: 1.39-2.91, *P* < 0.01) for patients divided by median value and 3.61 (95% CI: 2.17-6.02, *P* < 0.01) for patients divided by other value. In addition, subgroup analysis showed high HOTAIR expression predicted poor OS for CRC patients regardless of sample size (≤ 100 vs. > 100) and NOS score (< 7 vs. ≥ 7).

**Table 2:** Subgroup analysis of association between HOTAIR expression and OS in CRC.

Subgroup	No. of studies	Heterogeneity		HR	95% CI	P
		I^2^ (%)	P			
Country						
China	4	0.0	0.58	2.16	1.56-2.98	< 0.01
Other	2	0.0	0.79	5.25	2.41-11.47	< 0.01
Cut-off value						
Median	3	0.0	0.50	2.01	1.39-2.91	< 0.01
Other	3	0.0	0.45	3.61	2.17-6.02	< 0.01
Sample size						
≤ 100	3	63.0	0.07	3.15	1.34-7.37	0.01
> 100	3	0.0	0.65	2.52	1.63-3.90	< 0.01
NOS score						
< 7	2	0.0	0.66	1.90	1.30-2.79	< 0.01
≥ 7	4	0.0	0.65	3.69	2.28-5.98	< 0.01

**Abbreviations:** OS, overall survival; CRC, colorectal cancer; HR, hazard ratio; CI, confidence interval; NOS, Newcastle-Ottawa Scale.

There were two studies reporting the relationship between HOTAIR and RFS in CRC patients. The pooled result (HR = 1.97, 95% CI: 1.27-3.05, *P* < 0.01) showed significant correlation between high expression of HOTAIR and shorter RFS with minor heterogeneity (*I^2^* = 49.7%, *P* = 0.16, Figure 2B).

### 3.3 Association between HOTAIR and clinicopathological features in CRC

The performed meta-analysis showed that high expression of HOTAIR was significantly associated with venous invasion (OR = 2.53, 95% CI: 1.12-5.68, *P* = 0.02), advanced tumor infiltration (OR = 3.35, 95% CI: 1.34-8.42, *P* = 0.01) and distant metastasis (OR = 5.52, 95% CI: 1.22-25.01, *P* = 0.03). There was no observably statistical difference

between HOTAIR expression level and tumor size, differentiation, lymph node metastasis and TNM stage (Table 3).

**Table 3:** Relationship between HOTAIR expression and clinicopathological variables in CRC.

Variables	No. of studies	Heterogeneity		OR	95% CI	P
		I^2^ (%)	P			
Tumor size (cm, ≥ 5 vs. < 5)	2	95.6	< 0.01	1.32	0.08-22.87	0.85
Differentiation (Poor vs. Well and Moderate)	4	91.1	< 0.01	3.48	0.52-23.28	0.20
Venous invasion (Yes vs. No)	2	0.0	0.38	2.53	1.12-5.68	0.02
Tumor infiltration (T3 and T4 vs. T1 and T2)	2	0.0	0.75	3.35	1.34-8.42	0.01
Lymph node metastasis (Yes vs. No)	4	79.8	< 0.01	1.98	0.80-4.90	0.14
Distant metastasis (Yes vs. No)	3	73.2	0.03	5.52	1.22-25.01	0.03
TNM stage (III and IV vs. I and II)	3	88.3	< 0.01	1.94	0.47-8.10	0.36

**Abbreviations:** CRC, colorectal cancer; OR, odds ratio; CI, confidence interval; TNM, tumor-node-metastasis.

### 3.4 Sensitivity analysis and Publication bias

**Figure 3:**
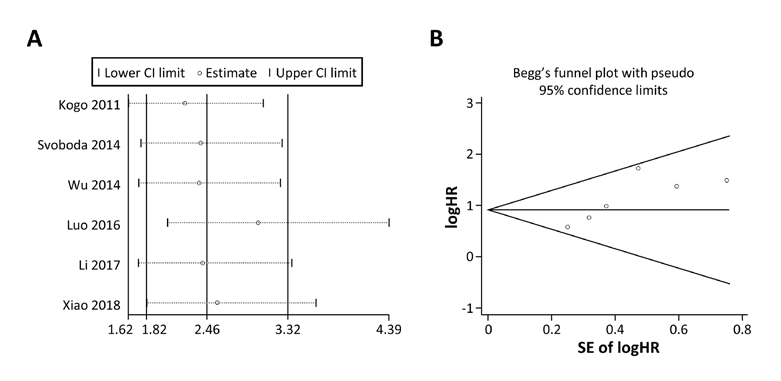
Sensitivity analysis for overall survival in colorectal cancer.

Sensitivity analysis for OS is displayed in Figure 3A. The results indicated that any of the included studies had little effect on the overall results, which suggested that our results were relatively stable and credible. Begg’s test and Egger’s test were employed to evaluate publication bias, showing that there was potential publication bias in OS (*P* = 0.13 for Begg’s test and *P* = 0.03 for Egger’s test, Figure 3B). Then, the trim and fill analysis was also performed, and after correction, the adjusted pooled HR was 2.05 (95% CI: 1.56-2.69, *P* < 0.01), which indicated that no significant publication bias existed.

### 3.5 Validation of the results in the TCGA database

**Figure 4:**
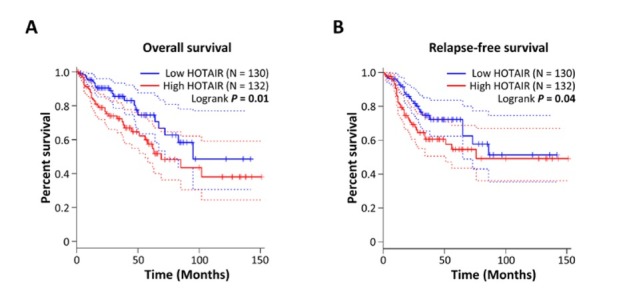
Validation the prognostic value of HOTAIR in the TCGA database. (A) HOTAIR and overall survival. (B) HOTAIR and relapse-free survival.

We first explored the relationship between HOTAIR expression and prognosis in CRC using the data from TCGA database. 262 CRC patients were extracted and then divided into high and low expression groups according to the median HOTAIR expression. The results suggested that high HOTAIR expression denoted a worse OS (*P* = 0.01, Figure 4A) and RFS (*P* = 0.04, Figure 4B) compared

with its low expression, validating that HOTAIR overexpression was significantly associated with OS and RFS in CRC patients.

## 4 Discussion

CRC is one of the most lethal malignancies and the fourth most frequently diagnosed cancer worldwide [1]. The high mortality rate and the overall poor survival in CRC patients indicate the importance of standardized treatment, predicting prognosis, and long-term follow-up [25, 26, 27]. It is thus crucial to identify prognostic biomarkers for CRC, which can help to develop clinical strategies and improve survival for CRC patients. Our study aimed to investigate the relationship between the HOTAIR expression and prognosis and clinicopathological features of CRC patients.

HOTAIR has been one of the most studied lncRNAs, which is a key regulator of chromatin states and dynamics by binding to the specific chromatin modification complex polycomb repressive complex 2 (PRC2) [28, 29, 30, 31]. It is associated with invasiveness, metastatic progression and poor prognosis in various cancers. Liu et al reported that HOTAIR was significantly up-regulated in non-small cell lung cancer (NSCLC) tissues and regulated invasion and metastasis by reducing homeodomain protein A5 or HOXA5, a tumor suppressor gene, in NSCLC cells [13]. A study indicated that HOTAIR was higher in hepatocellular carcinoma tissues than that in adjacent normal tissues, and significantly associated with poor differentiation, metastasis, progression and prognosis [15]. A clinical study suggested that high HOTAIR expression in breast cancer was significantly associated with poor prognosis, particularly in patients with estrogen receptor (ER)-positive tumors [32]. High HOTAIR expression was also used as a predictor of poor OS in gastric cancer (GC), and inhibition of HOTAIR could reduce invasiveness and reverse epithelial-mesenchymal transition (EMT) in GC cells [14].

The present meta-analysis involved 6 studies and revealed that high expression of HOTAIR was significantly associated with poor OS and RFS in CRC patients. Previous studies revealed that HOTAIR overexpression could predict unfavorable outcome in gastric cancer [33,34]. Another meta-analysis showed there was a significant association between high expression of HOTAIR and poor OS in patients with digestive cancers [35,36]. Liu et al reported that HOTAIR expression was significantly increased in cancer tissues compared with that in normal tissues, and its expression level was a risk factor for OS in patients with cervical cancer [37]. Several other studies also found similar results [38, 39, 40]. Moreover, there was an obvious relationship between high expression of HOTAIR and clinicopathological parameters, such as venous invasion, advanced tumor infiltration and distant metastasis. Then we employed the TCGA database to investigate the prognostic value of HOTAIR in CRC, with the results indicating that HOTAIR may serve as a reliable biomarker for the prognosis of CRC patients. More and more studies have been conducted to explore the mechanism of HOTAIR in the pathogenesis of CRC, but there is still no clear conclusion. Li et al. showed that HOTAIR contributed to 5FU resistance through suppressing miR-218 and activating NF-κB/TS signaling in CRC [17]. HOTAIR also regulated the progression and chemoresistance of CRC via modulating the expression levels of miR-203a-3p and the activity of Wnt/β-catenin signaling pathway [24]. In addition, HOTAIR could elicit an inhibitory effect on proliferation, invasion, and migration, while promoting the apoptosis of CRC cells through the upregulation of p21 [41].

There are some limitations in this meta-analysis. First, only English papers were included in the present study, which may exclude potentially relevant articles. Second, some of the studies only showed Kaplan-Meier curves without HRs and CIs, meaning that survival curves had been reconstructed to extract data and calculate the HRs and CIs. Third, there were no consensus about cutoff values for high and low HOTAIR expression among studies. Therefore, we conducted subgroup analysis according to cut-off values and the pooled results confirmed the prognostic value of HOTAIR in CRC patients, which indicated that the difference of cut-off values did not affect the stability of the results. Moreover, the funnel plot analysis of OS showed some asymmetry and publication bias was confirmed by Begg’s test and Egger’s test. Thus, the pooled result may be somehow overvalued. But the followed trim and fill analysis did not change the overall result, which further reinforced the prognostic value of HOTAIR in CRC patients.

In summary, high expression of HOTAIR was significantly associated with poor OS and RFS, indicating that HOTAIR could serve as a reliable prognostic biomarker for CRC patients. Further, high HOTAIR expression was found to be associated with venous invasion, advanced tumor infiltration and distant metastasis.
